# Symptom Profile and Severity in a Sample of Nigerians with Psychotic versus Nonpsychotic Major Depression

**DOI:** 10.1155/2013/815456

**Published:** 2013-08-21

**Authors:** Increase Ibukun Adeosun, Oyetayo Jeje

**Affiliations:** Federal Neuropsychiatric Hospital, Yaba, 8 Harvey Road, PMB 2008, Lagos, Nigeria

## Abstract

The therapeutic strategies in managing patients with psychotic major depression (PMD) differ from those with non-psychotic major depression (NMD), because of differences in clinical profile and outcome. However, there is underrecognition of psychotic symptoms in depressed patients. Previous studies in Western population suggest that certain symptom patterns, apart from psychosis which may be concealed, can facilitate the discrimination of PMD from NMD. These studies may have limited applicability to sub-Saharan Africa due to cross-cultural differences in the phenomenology of depression. This study compared the rates and severity of depressive symptoms in outpatients with PMD (*n* = 129) and NMD (*n* = 117) using the Structured Clinical Interview for Depression (SCID) and Hamilton Depression Rating Scale (HAM-D). Patients with PMD had statistically significantly higher rates of suicidal ideation, suicidal attempt, psychomotor agitation, insomnia, and reduced appetite. Patients with NMD were more likely to manifest psychomotor retardation and somatic symptoms. PMD was associated with greater symptom severity. On logistic regression analysis, suicidal ideation, psychomotor disturbances, insomnia, and somatic symptoms were predictive of diagnostic status. The presence of these symptoms clusters may increase the suspicion of occult psychosis in patients with depression, thereby informing appropriate intervention strategies.

## 1. Introduction

Depression is a common psychiatric disorder estimated to affect about 1 out of 5 people in their lifetime [1]. It is a leading cause of disability, projected to become the second leading contributor to the global burden of disease by the year 2020 [2]. Clinically, a major depressive episode or disorder is characterised by the presence of depressed mood, loss of interest, low energy, and significant changes in sleep, appetite, weight, and psychomotor activity. Other symptoms include indecisiveness, feelings of worthlessness, guilt, and suicidality [3, 4].

 Epidemiological evidence indicates that about 19 to 25% of patients with major depression have psychotic symptoms, typically delusions and hallucinations [5]. In comparison to Nonpsychotic major depression (NMD), patients with psychotic major depression (PMD) have a worse clinical course characterised by higher rates of recurrence, treatment resistance, suicidality, psychosocial impairment, overall symptom severity, and comorbidity [6–9]. 

 Research has shown that there is underdetection of psychotic symptoms in depressed patients because psychotic symptoms may be subtle, intermittent, underreported, or deliberately concealed due to fear of embarrassment or stigma [10]. Considering the worse outcome in PMD, it is critical to identify features that could facilitate the clinical discrimination of PMD from NMD in addition to the presence or absence of psychotic symptoms. 

Furthermore, the common notion that the severity of symptoms is an index of the presence of psychosis in depressed patients has been challenged by recent research findings indicating that psychosis is not inextricably linked to a higher severity of depressive symptoms [5, 7]. 

The majority of previous studies comparing the pattern and severity of symptoms in PMD versus NMD found that depressed patients with psychosis were more likely to manifest insomnia, psychomotor agitation, guilt, cognitive impairment, suicidal ideation, and suicidal attempt [10–14]. PMD was also associated with greater severity of depressive symptoms. Findings regarding differences in the prevalence and severity of anxiety, diurnal variation, hypochondriasis, and depressed mood are less consistent [15]. Forty et al. [11] found wide variations in severity in both patients with PMD and NMD, and showed that severity alone is not the determinant of psychosis in patients with depression. Rates of up to 15% of psychosis have been reported in patients with mild to moderate level of severity of depressive symptoms [5].

 Previous authors have reported the phenomenology and correlates of depression in Africans, as well as certain differences between unipolar and bipolar depression [16–24]. However, there is limited information on the symptom and severity profile of patients with PMD in comparison to NMD using standardised instruments. Cross-cultural differences in the phenomenology of depression may limit the applicability of findings from Western countries to a sub-Saharan African population [25]. For instance, depression is more likely to be manifestd with somatic symptoms in Africans, whereas symptoms such as guilt, worthlessness, and suicidality common in Caucasians may occur with less frequency [5, 16–25]. Furthermore, ethnocultural differences are known to exist in the prevalence and manifestation of psychotic symptoms [26]. On account of these, previous authors have highlighted the need to examine the presentation of PMD and NMD from diverse ethnic and socio-cultural settings [12, 27].

 This study therefore aimed to compare the rates and severity of symptoms of depression between patients with PMD and those with NMD among out-patients attending a major psychiatric care facility in Nigeria. Elucidations of these differences have implications for accurate diagnosis, timely intervention, and treatment planning. Based on the extant literature, we hypothesised that there are significant differences between PMD and NMD in frequency and severity of depressive symptoms.

## 2. Materials and Methods

### 2.1. Subjects

 Consecutively presenting out-patients (*n* = 246) with PMD and NMD attending the largest mental health care facility in Nigeria, Federal Neuro-Psychiatric Hospital Yaba, were recruited into the sample over a period of four months (from January to April 2012). Exclusion criteria were subjects below the age of 18 years, the presence of comorbid medical/neurological disorders, mood/psychotic disorders due to a general medical condition, and other comorbid psychiatric disorders.

### 2.2. Procedure

 Ethical approval was obtained from the institution's research and ethical committee. Consecutively presenting patients were invited to participate in the study and subjects were recruited following the provision of informed consent. There were only seven refusals and these did not differ significantly from the recruited sample in terms of demographic characteristics. Patients were recruited at the study setting following comprehensive psychiatric and medical assessment by consultant psychiatrists and senior residents in psychiatry. They were recruited at first contact with the facility prior to commencement of treatment. The researchers had access to patients clinical records (case notes) and in most cases collateral information by accompanying family members. Clinical diagnoses of depression were ascertained with the Structured Clinical Interview for DSM-1V Axis I Disorder (SCID) [28]. Comparisons were made between patients with PMD and NMD regarding the prevalence and severity of symptoms of depression using the SCID and the Hamilton Depression Rating Scale (HAM-D) [29], respectively. Interviewer-based instruments were completed by trained researchers with a minimum of 2-year postgraduate residency training in psychiatry. Interrater assessments from 10 joint interviews during a pretest yielded reliability coefficients, *K* = 0.90 for depression (SCID), *K* = 0.88 for “other somatic symptoms,” and *r* = 0.89 for HAM-D.

### 2.3. Measures

#### 2.3.1. Sociodemographic Questionnaire

The questionnaire was designed by the authors to elicit information about the age, gender, marital status, highest level of education and employment status of the participants. The literate participants completed the questionnaire by themselves. The researchers read out the questions and response options to those who were not literate and recorded the responses they volunteered.

#### 2.3.2. SCID

The Structured Clinical Interview for DSM-1V Axis I Disorder (SCID) was administered to ascertain the diagnosis of depressive disorder (PMD and MD) in the participants. It also provided rating of symptoms of depression ranging from 1 (none), 2 (subthreshold), to 3 (threshold). Depressive symptoms were regarded as present (positive) only if the response items were rated as 3. In addition to the symptom profile elicited with the SCID, we also included the item “other somatic symptoms” in order to capture certain somatic symptoms (such as heat or peppery sensations in the head or body; heaviness or tension in the head; emptiness or feelings of fluid in the head; and crawling sensations) which have been shown by previous authors to be common or uniquely expressed among Africans [20–24]. In view of the fact that the expression of these symptoms may be related to the cultural idioms of distress or the use of somatic metaphors [25], “other somatic symptoms” were painstakingly probed clinically to ascertain their nature, quality, and frequency. For instance, patients were asked to describe these symptoms in detail including the use of local language in order to clarify if symptoms such as heat/pepper/crawling sensation were merely somatic metaphors or met the definition of tactile hallucinations or somatic delusions. When the symptoms are accompanied by delusional interpretation/explanations or the patients have clear somatic delusions or tactile hallucination (not merely a somatic metaphor) on further probing, these are regarded as psychotic symptoms. On the other hand if they were only idiomatic expressions or pseudo-hallucination occurring exclusively during mood episodes, they were regarded as nonpsychotic symptoms.

#### 2.3.3. Hamilton Depression Rating Scale (HAM-D)

It was administered by the researcher to rate the severity of depressive symptoms in the participants. It is a 21-item interviewer based instrument, but only the first 17 items responses were summed up to determine the severity of depressive symptoms.

### 2.4. Statistical Analyses

Data was analysed with the SPSS version 16. The sociodemographic and clinic characteristics were compared using chi-square for categorical variables and *t*-test for continuous variables. The primary outcome of interest was the presence or absence of symptoms of depression in patients with PMD versus NMD. This was compared using chi-square. Group comparison of symptom severity (mean total HAM-D) score was conducted with the independent Student's *t*-tests. To test for independent significant relationships, significant variables on bivariate analysis were all entered into logistic regression analysis. The effects of difference in severity were controlled for. The test was essentially two-tailed with level of significance set at *P* < 0.05. 

## 3. Results

Table 1 shows the socio-demographic profile of the participants. Females were the majority (66.3%) and their mean age was 39.9 (±13.3) years. Only 30.5% had up to tertiary education, 59.3% were unemployed, and 58.5% were married. There were no significant socio-demographic differences between patients with PMD and NMD.

As compared with patients with NMD, patients with PMD had significantly higher rates (Table 2) of psychomotor agitation (*P* < 0.001), insomnia (*P* < 0.001), reduced appetite (*P* = 0.038), suicidal ideation (*P* = 0.005), and suicidal attempts (*P* = 0.009). On the other hand, the rates of “other somatic symptoms” and psychomotor retardation were significantly higher in patients with NMD (*P* < 0.001). Similarly patients with PMD had significantly higher overall depression severity rating in comparison to patients with NMD (*P* < 0.001). 

The most common psychotic symptoms in patients with PMD were auditory hallucinations (54.26%), persecutory delusions (42.64%), and delusions of reference (33.33%). Others included delusions of guilt, religious delusions, visual hallucinations, somatic delusions, tactile hallucinations, and delusions of jealousy (Table 3).

On regression analysis, psychomotor agitation, suicidal ideation, and insomnia were independently associated with PMD, while somatic symptoms and psychomotor retardation were predictive of NMD (Table 4).

## 4. Discussion

This study compared the frequency and severity of depressive symptoms between patients with PMD and NMD in an out-patient psychiatric setting in Nigeria. The prevalence of PMD in the current study sample is higher than those of previous studies in Western populations. Previous authors in Western populations reported that the prevalence of psychotic symptoms in patients with depression is about 19% in community samples and 25% in psychiatric hospital settings [5, 12, 30]. The higher prevalence of PMD in our sample may be attributed to difference in the pattern of service use across settings. Due to low levels of mental health literacy, causal misattribution, and poor access to mental health services in Nigeria, it is plausible that patients are more likely to seek treatment for more severe or psychotic symptoms. In a consecutive sample of out-patient clinic attendees in a similar setting to ours, 61% of depressed patients presenting to services had severe depression [16, 17]. Furthermore, previous studies have documented racial differences in the prevalence and expression of psychotic features in depressed patients [26, 27]. The most common psychotic symptoms in the current sample were auditory hallucinations, persecutory delusions, and delusions of reference. Previous studies have shown that the psychotic symptoms commonly seen in patients with depression are auditory hallucination, paranoid delusions, somatic delusions, delusions of guilt, and tactile hallucinations. The prevalence of tactile hallucination appears lower than that reported among depressed patients in Western population [12, 13].

We found that the symptom profile of patients with psychotic depression differed significantly from that of patients with nonpsychotic depression. This is in keeping with previous research [12, 13, 30, 31]. Our findings of a significantly higher rates of psychomotor agitation, insomnia, and suicidal ideation and attempt in patients with PMD in comparison to NMD are congruent with these reported by Guadiano et al. [12] in a large sample of outpatients with psychotic and nonpsychotic depression in the USA. Other authors have also demonstrated higher prevalence of psychomotor disturbances [13, 15, 32], insomnia [25], and suicidality [13, 22, 33] in patients with PMD. 

Patients with PMD had significantly higher rates of reduced appetite than patients with NMD. Previous studies differed regarding this finding. Some authors reported that patients with PMD were more likely to have reduced appetite, [11] while other authors found no significant difference between both groups [12]. In contrast to previous studies, the prevalence of feelings of guilt and worthlessness did not significantly differ between PMD and NMD in the current study. The lack of significant difference in the rates of dysfunctional cognitions between both groups could be attributed to the lower frequency of these symptoms in the current sample, which is not surprising considering the documented rarity of guilt and worthlessness in the phenomenology of depression among Africans [16, 17].

 On the other hand, patients with PMD had significantly lower rates of “other somatic symptoms” than those with NMD. Somatic symptoms are commonly expressed by Nigerian patients with depression [20–24]. Ilechukwu [20] found that somatic symptoms were more common in mild and moderate depression than severe depression in a Nigerian clinical population. However, to the best of our knowledge, these somatic symptoms have not been previously shown to be predictive of either PMD or NMD among Africans. Future studies are required to confirm this finding.

 The elucidation of patterns of symptoms that are more likely to be associated with PMD, apart from psychotic symptoms which may be underreported, is of clinical significance [10]. Clinician's recognition of these patterns of symptoms could help in overcoming the diagnostic conundrum involved in differentiating PMD from MD. Early identification of PMD is crucial in treatment planning considering its worse clinical course and outcome. 

Another important finding is the significantly higher level of symptom severity in PMD than NMD. This reechoes the findings of several authors [12, 31, 33] and reflects the definition of PMD on the basis of severity. However, studies have shown that higher depression scores in PMD are attributable to specific rather than global symptom elevation and greater illness severity is not a specific clinical marker for the presence of psychotic symptoms in patients with depression [11, 34]. Keller et al. [35] noted that the inextricable link between severity and psychosis is a pitfall; they emphasised the need to revise the classification of PMD, which is currently under the severity dimension. 

On account of these vital differences in PMD and NMD, which have also been substantiated with neurochemical correlates, it has been argued that PMD is a distinct syndrome from NMD rather than a continuum of NMD manifesting with a greater severity [34–36]. Beyond issues of controversies regarding the classification, of more importance is the clinical/therapeutic significance of these findings. Since PMD responds poorly to conventional antidepressants and is more likely to be associated with resistance to treatment, prompt delineation of PMD from MD will inform better treatment planning [33].

 Our study was hospital based and may not be representative of the profile of patients with depression in the general population. We do not know to what extent our cultural inclination or biases affected our clinical interpretation of the symptoms defined as “other somatic symptoms” and how these may apply in other settings. Our findings may also be limited by differences in certain variables such as age of onset of illness, family history, number of episodes of depression, and impact of medications. The latter two factors may be minimal due to the fact that patients were recruited at first contact with psychiatric services and therefore expectedly neuroleptic-naive. However, in the study setting, many patients present late for treatment and the possibility of undisclosed self-medication or unlabelled prescribed medication from other sources cannot be totally ruled out. Though patients with comorbid psychiatric disorders were excluded, due to the cross-sectional nature of the study, it is not known if patients presenting with a depressive episode may eventually evolve to other mood disorder (e.g., bipolar disorder) if followed up longitudinally; this is important considering the evidence that the best predictor of psychosis in the course of mood disorder is bipolar disorder [33]. However, this study has a number of strengths; we provided data from a previously understudied ethnic and cultural setting therefore filling a gap in knowledge. Secondly, participants were also included regardless of their gender and level of education. Thirdly, standardised instruments were used in the assessment of the presence and severity of symptoms. Furthermore, the impact of medications may be minimal because patients were recruited at first contact with services. The availability of collateral information from clinical records and informants is also an improvement on previous studies. 

## 5. Conclusion

 In conclusion, the findings of the current study, conducted in a previously understudied ethnic and socio-cultural context, is consistent with previous findings that certain cluster of symptoms may increase the suspicion of occult psychosis in patients with depression. Clinicians should have high index of suspicion of PMD when depression is manifested by psychomotor agitation, insomnia, suicidality, and absence of somatic symptoms. Further studies are required to confirm these findings in our environment. Future studies should consider longitudinal assessments of symptoms in a larger sample. 

## Figures and Tables

**Table 1:** Socio-demographic characteristics of the participants.

Variables	PMD (*n* = 129) *n* (%)	NMD (*n* = 117) *n* (%)	Total	*χ* ^2^	*P*
Gender					
Male	38 (29.5)	46 (39.3)	84	2.652	0.103
Female	91 (70.5)	71 (60.7)	162		
Marital status					
Married	83 (64.3)	62 (53.0)	145	3.266	0.071
Not married	46 (35.7)	55 (47.0)	101		
Employment status					
Employed	56 (43.4)	44 (37.6)	100	0.857	0.355
Unemployed	73 (56.6)	73 (62.4)	146		
Level of education					
Primary/none	38 (29.5)	22 (18.8)	60	4.066	0.131
Secondary	53 (41.1)	59 (50.4)	112		
Tertiary	38 (29.5)	36 (30.8)	74		

	Mean (SD)	Mean (SD)		*t*	

Age	39.94 (13.47)	39.85 (13.22)		0.051	0.960

**Table 2:** Prevalence of symptoms of depression in patients with PMD versus NMD.

Symptoms	PMD (*n* = 129) *n* (%)	NMD (*n* = 117) *n* (%)	*χ* ^2^	*P*
Depressed mood	129 (100)	116 (99.1)	1.11*	0.476
Diminished interest	108 (83.7)	95 (81.2)	0.27	0.603
Decreased appetite	70 (54.3)	48 (41.0)	4.31	0.038
Weight loss	41 (31.8)	35 (29.9)	0.10	0.751
Insomnia	127 (98.4)	93 (79.5)	23.34	**<0.001**
Psychomotor agitation	41 (31.8)	10 (8.5)	20.16	**<0.001**
Psychomotor retardation	24 (18.6)	53 (45.3)	20.33	**<0.001**
Fatigue/low energy	98 (76.0)	93 (79.5)	0.44	0.508
Guilt	51 (39.5)	43 (36.8)	0.20	0.654
Worthlessness	63 (48.8)	65 (55.6)	1.11	0.292
Poor concentration	64 (49.6)	56 (47.9)	0.08	0.784
Suicidal ideation	75 (58.1)	47 (40.2)	7.92	**0.005**
Suicidal attempt	23 (17.8)	8 (6.8)	6.73	**0.009**
Other somatic symptoms	38 (29.5)	64 (54.7)	16.11	**<0.001**

HAM-D score	Mean (SD)	Mean (SD)	*t*	*P*

HAM-D score	27.64 (3.9)	18.64 (6.1)	13.93	<0.001

*Fisher's correction applied.

**Table 3:** Profile of psychotic symptoms in patients with psychotic major depression (*N* = 129).

Symptoms	*n* (%)
Delusions	
Persecution	55 (42.64)
Reference	43 (33.33)
Guilt	10 (7.75)
Religion	8 (6.20)
Somatic	7 (5.43)
Jealousy	3 (2.32)
Hallucinations	
Auditory	70 (54.26)
Visual	8 (6.20)
Tactile	7 (5.43)

**Table 4:** Logistic regression analysis of depressive symptoms pattern in PMD versus NMD.

Variables	*B*	SE	Wald	*P*	OR	95% CI
Psychomotor agitation	.95	.42	5.02	0.025	2.60	1.13–5.96
Psychomotor retardation	−1.29	.34	14.56	<0.001	0.28	0.14–0.54
Insomnia	2.70	.79	11.59	0.001	14.90	3.15–7.57
Decreased appetite	.11	.31	0.13	0.715	1.12	0.61–2.04
Other somatic symptoms	−0.69	.32	4.56	0.033	0.50	0.27–0.94
Suicidal ideation	.86	.33	6.58	0.010	2.36	1.22–4.53
Suicidal attempt	.54	.54	1.00	0.317	1.72	0.59–5.01
Symptom severity	1.66	.81	4.21	0.041	2.54	1.07–4.21

OR: odds ratio.
